# The role of executive function in the processing and acquisition of syntax

**DOI:** 10.1098/rsos.201497

**Published:** 2025-03-27

**Authors:** Malathi Thothathiri, Evan Kidd, Caroline Rowland

**Affiliations:** ^1^ Department of Speech, Language and Hearing Sciences, The George Washington University, Washington, DC, USA; ^2^ Max Planck Institute for Psycholinguistics, Nijmegen, Gelderland, The Netherlands; ^3^ Research School of Psychology, Australian National University, Canberra, Australian Capital Territory, Australia; ^4^ ARC Centre of Excellence for the Dynamics of Language, Australian National University, Canberra, Australian Capital Territory, Australia; ^5^ Donders Institute for Brain, Cognition and Behaviour, Radboud Universiteit, Nijmegen, Gelderland, The Netherlands

**Keywords:** executive function, syntax, eye-tracking, children, sentence processing, language acquisition

## Abstract

Language acquisition is multifaceted, relying on cognitive and social abilities in addition to language-specific skills. We hypothesized that executive function (EF) may assist language development by enabling children to revise misinterpretations during online processing, encode language input more accurately and/or learn non-canonical sentence structures like the passive better over time. One hundred and twenty Dutch preschoolers each completed three sessions of testing (pre-test, exposure and post-test). During pre-test and post-test, we measured their comprehension of passive sentences and performance in three EF tasks. In the exposure session, we tracked children’s eye movements as they listened to passive (and other) sentences. Each child was also assessed for short-term memory and receptive language. Multiple regression evaluated the relationship between EF and online processing and longer-term learning. EF predicted online revision accuracy, while controlling for receptive language, prior passive knowledge and short-term memory, consistent with theories linking EF to the revision of misinterpretations. EF was also associated with longer-term learning, but the results could not disentangle EF from receptive language. These findings broadly support a role for EF in language acquisition, including a specific role in revision during sentence processing and potentially other roles that depend on reciprocal interaction between EF and receptive language.

## 1. Introduction

A key aspect of language comprehension is to understand events and scenarios described by others. For example, when hearing an active sentence (e.g. *The boy was chasing the dog*), we must infer that the boy was the chaser. In contrast, for a passive sentence (e.g. *The boy was chased by the dog*), we must understand that the boy was the entity being chased rather than the one doing the chasing. Young children acquiring English show early comprehension of actives beginning in the second year of life, but their understanding of passives lags significantly behind, even extending to school years (see [1,2] and references therein). More broadly, children show earlier facility with typical or canonical word orders (e.g. actives, subject relative clauses) than non-canonical word orders (e.g. passives, object relative clauses) [3]. Studies of online sentence processing have suggested that children use parsing heuristics that are biased towards more frequent word orders [1,2], which could explain the observed acquisition pattern. In the case of the active/passive alternation, a tendency to interpret the first noun as an agent leads to processing difficulty for passives. This has been observed in adult language processing (e.g. [4]) but appears particularly problematic in acquisition because children regularly fail to revise their initial (mis)interpretation [1,5–8].

Beginning with Novick *et al*. [9], it has been suggested that failures to revise during comprehension might be linked to executive functioning, which is used to regulate behaviour more broadly. Executive function (EF) comprises a complex set of skills, including inhibition, attentional regulation, set-shifting and planning. These abilities are supported by frontal regions of the brain, which undergo a protracted period of maturation in children [10]. Preschoolers show gradual improvement in their executive functioning [11–13], and thus one hypothesis is that the development of EF skills is linked to the ability to revise initial parsing commitments [6,8,14]. However, empirical evidence is relatively scarce and has thus far been limited to syntactically ambiguous, garden-path sentences [15,16]. Furthermore, prior investigations were restricted to processing and did not address the consequences (if any) for acquisition. The present study sought to fill these gaps. We articulated and tested hypotheses about the impact of EF on the acquisition of non-canonical sentence structures using an individual differences approach in 120 preschool children.

We begin by reviewing: (i) previous studies on online sentence processing in children, and (ii) prior evidence for a link between EF and sentence comprehension in adults and children, before turning to the present study.

### 1.1. Prechoolers’ online sentence processing

Trueswell *et al*. [7] first demonstrated that 5-year-old children struggle to revise their ongoing interpretation of sentences like (1).

(1) *Put the frog on the napkin in the box*.

Children initially interpreted ‘on the napkin’ as a destination for the frog and unlike adults, they failed to correct that misinterpretation after encountering the disambiguating phrase ‘in the box’, which indicates that ‘on the napkin’ should be construed as a modifier (which frog) rather than as a destination. Multiple other studies have replicated this effect using similar and even simpler materials [5,6,8]. The clear consensus from these studies is that children, like adults, process sentences incrementally, constructing the meaning of a sentence moment-to-moment as it unfolds. While such rapid incremental comprehension could be efficient and advantageous under many situations, it can lead to misinterpretation when the syntax is temporarily ambiguous and resolves towards a less common meaning (e.g. the modifier interpretation in (1)). In such cases, the original interpretation must be revised when later portions of the sentence disambiguate the syntax. Adults show detectable signs of garden-pathing but eventually arrive at the correct meaning a majority of the time (e.g. see [7]). Preschool children, by contrast, struggle with revision—for example, they act out sentences like (1) by first putting the frog on the napkin and then putting it in the box, or ignoring the box entirely [5,7,8].

Failure to revise an original interpretation could also impact children’s comprehension of unambiguous sentences with non-canonical word order. In particular, it has been suggested that during the incremental processing of transitive sentences, both adults and children might use a "first noun phrase (NP) is agent" heuristic, which would provide an initial meaning that turns out to be correct for an active sentence (e.g. *The boy was chasing the dog*, where the boy is ultimately the agent) but not a passive sentence (e.g. *The boy was chased by the dog*, where the boy is ultimately the patient) [4,17,18]. Difficulty with revision would therefore impact passives more than actives. Consistent with this account, Huang *et al*. [2] found that 5-year-old children were more likely to misinterpret Mandarin passives as actives if the passive marker appeared after a referential noun instead of before it. They concluded that once a referential noun had been construed as the agent of an action, it might be hard for children to revise that construal. Similarly, Abbot-Smith *et al*. [1] found that younger 2 and 3-year-old children’s online interpretation of passives containing novel verbs showed a predilection for initially mapping the first noun in the sentence to the agent role. Analysis of offline choices further suggested that the younger age group could not successfully overcome that initial mapping.

Overall, considerable evidence supports the idea that children, like adults, process sentences incrementally. However, their ability to revise these incremental interpretations based on later arriving sentence content is more fragile. Why might this be the case? While early online comprehension studies with children suggested a role for a non-specific processing or memory span limitation [5,7], later papers have postulated a specific role for EF [1,2,8,14] based primarily on neuroimaging, neuropsychological and behavioural evidence from adults that is tentatively supported by sparser evidence from children. We turn to this set of findings next.

### 1.2. Link between executive function and sentence comprehension

Multiple neuroimaging studies with adults have found that left frontal regions associated with EF are also engaged during the comprehension of sentences that generate conflict between alternative interpretations, including garden-path sentences [19–22]. Neuropsychological evidence from aphasia further suggests an association between damage to the left frontal cortex and difficulty in overcoming prior processing biases [23,24]. Training or activating EF improves the processing of sentences containing conflict, suggesting a causal relationship [25–27]. Altogether, there is considerable evidence that EF is relevant for sentence comprehension in adults, especially in cases requiring the revision of an original interpretation or selection between alternatives.

The evidence from adults provides prima facie support for the proposal that immature EF might be one reason why children struggle to revise their interpretation of garden-path and non-canonical sentences. To our knowledge, only two known studies have directly examined the link between the two in typically developing preschoolers [15,16] (see also [28]). For related evidence in other language domains, see Khanna & Boland [29] and Nilsen & Graham [30]. Woodard *et al*. [16] reported that better performance in switching between rules (as measured via a Flanker/No-Go task) corresponded with better garden-path recovery in 5-year-old children. Children with better EF scores committed fewer errors in acting out ambiguous, garden-path sentences. Similarly, Qi *et al*. [15] found a relationship between children’s scores on two EF tasks (Simon Says and Flanker/Reverse Flanker) and their accuracy for syntactically ambiguous sentences. Both of these studies investigated children’s processing of sentences like (1).

To date, no study has examined the relationship between individual differences in EF and the reanalysis of unambiguous non-canonical sentences like the passives. A recent clinically oriented study looked at how typically developing children and children with developmental language disorder comprehend canonical and non-canonical sentences (including passives), and whether individual differences in abilities like fluid reasoning, controlled attention, complex working memory and language knowledge explain variation in children’s language comprehension [31]. Children were asked to identify the agent in highly implausible, semantically reversible sentences (e.g. *The train was watched by the bed under the cold cake*). The authors reported that in both populations, complex working memory mediated the relationship between the other abilities (i.e. fluid reasoning, controlled attention and language knowledge) and sentence comprehension.

A distinguishing feature of the current study is process-specificity. We propose that EF, particularly the selection of a representation or response from among multiple competing alternatives, is useful specifically for revising the initial misinterpretation of sentences during online processing (see more below). This approach differs from the examination of broader, less specific effects of other cognitive abilities on language. Skills like fluid reasoning and long-term memory could impact how children attend to language input, observe patterns and store the results of their analysis (see, e.g. [31]). Similarly, EF, writ large, could be construed to include skills such as analogical reasoning and awareness that could play a role in language acquisition. The hypotheses tested in the present study are more theoretically specific. We seek to understand if and how selection between competing alternatives, in particular, influences the comprehension of passive sentences in a process-specific manner that is grounded in psycholinguistic theories of incremental sentence processing. The results therefore are better positioned to help inform and refine those theories. In taking this approach, we extend prior investigation of children’s garden-path sentence processing [15,16] to the processing of unambiguous non-canonical sentences like the passives. Furthermore, we investigate the consequences of EF for the acquisition (cf. processing) of non-canonical syntax, which has not been explored before.

### 1.3. The present study

We assessed Dutch-speaking 4- and 5-year-old children’s comprehension of the passive structure in a pre-test–exposure–post-test design. This age group shows developing, not yet mature knowledge of passives (see, e.g. [32]), that can improve with further exposure to passive sentences, making it suitable for testing our theoretical claims (see below). Children of this age have also been well-studied for their ability to complete several language comprehension and EF tasks, making the investigation empirically more viable than it would be with younger 2- or 3-year-old children. During the pre- and post-test sessions, participants completed an offline sentence comprehension test measuring their knowledge of passives. In between, they underwent an exposure session that provided input containing passive sentences and used eye-tracking to assess online processing. In addition to these sentence tasks, children completed three EF tasks (Day/Night, Go-No-Go and Flanker/Reverse Flanker), a short-term memory task (Word Span) and a standardized assessment of receptive language (Schlichting test [33]).

Our reasoning consisted of two premises:

Pr1: EF will assist in children’s processing of passive sentences because children have a first-NP-is-agent bias that must be revised for accurate comprehension of passives. As described above, considerable evidence in adults and tentative evidence in children has linked EF to the revision of sentence interpretations. Therefore, we expected individual differences in children’s EF to predict their online revision of passive sentences, as indexed by eye movement measures during the middle exposure session.

Pr2: EF will indirectly assist in children’s acquisition of passives because it facilitates the accurate processing of passive sentences heard in the input (per (a)). First, we predicted that individual differences in children’s revision of online interpretations would be correlated with how much their comprehension of passives improves from pre-test to post-test. Put another way, we expected children with better revision to learn more from the passive input received during the exposure session. Second, we predicted that EF, by virtue of its theorized link to revision, would also be correlated with improvement in the comprehension of passives.

The analyses (see §2) tested the predictions arising from Pr1 and Pr2 by evaluating the relationship between passive sentence comprehension, revision and different EF measures. Several considerations guided our choice of specific EF tasks. We first chose the Day/Night task [34] because it is widely regarded as a child-appropriate analogue of the colour-word Stroop task that has been used with literate adults in a majority of the relevant adult literature (e.g. [20,22,24,25,27]). This task measures *inhibition*, which is thought to be especially relevant for suppressing a predominant sentence interpretation to choose an alternative meaning [9]. Measurements of EF are subject to different practical and conceptual issues, however. Psychometric data are sparse and mixed, with some studies suggesting that the Day/Night task has acceptable internal consistency and test–retest reliability (see [35] and references therein) and others reporting only moderate to poor reliability [36]. Aggregating across tasks can improve the reliability of measuring individual differences [36]. Therefore, we chose two additional paradigms that are also widely used with young children and involve inhibition, namely Go/No-Go and Flanker/Reverse Flanker [37,38].

Conceptually, the presence of different EF components and the relationship between them has not yet been fully determined. In adults, three separable components— inhibition, updating and set-shifting—have been proposed [39]. However, it is important to note that the list might not be limited to these components [40]. Additionally, the hypothesized components are strongly correlated even in adults and appear to be even less differentiable in young children [41]. Aggregating across tasks that share a common component, as described above, is consistent with evidence suggesting that EF might be a unitary construct in preschoolers [41]. For this reason, we use the broader ‘executive function’ label throughout this paper instead of the more specific ‘cognitive control’ label that is typically used in the relevant adult literature. However, our use of ‘executive function’ here is still specific to operations dealing with the handling of competing representations, responses and rules. In our stated hypotheses and interpretations, the term does not include relational reasoning, metalinguistic awareness and other skills that are often subsumed under the same label.

Our analysis strategy reflected the considerations above. We computed an aggregated score by averaging the *z* scores from three standard EF tasks, all of which involved inhibition and/or selection between competing alternatives, and used that as a predictor variable to test if EF explained variation in the processing and acquisition of passive structures. Across all analyses, we included pre-test, receptive language and short-term memory scores as control variables that could show individual differences that impact passive sentence comprehension but are theoretically distinct from the construct of EF. The results can shed light on persistent debates in the field. First, whether non-syntax-specific skills such as those measured by the EF tasks used here can influence syntactic processing and acquisition at all would be questioned under theories assuming language modularity (e.g. [42]). On a more fine-grained level, because some populations (e.g. people with aphasia) show normal online processing but impaired offline comprehension accuracy, it is possible that the former proceeds autonomously and that only the latter is subject to influences from EF on end-of-sentence integration and decision-making [43,44]. By testing the relationship between EF and online sentence processing and longer-term acquisition in young children, this study can offer important new evidence on the relationship between language and other cognitive functions early in development when naturalistic language processing can be queried with relatively little contamination from task-based strategies.

## 2. Methods

### 2.1. Participants

One hundred and twenty (*n* = 120) typically developing 4- and 5-year-old children who were native Dutch speakers participated. They were recruited under a protocol approved by the Ethics Committee of the Faculty of Social Sciences at Radboud University. The inclusion criteria for recruitment were as follows: (i) no diagnosed developmental or acquired disorder that impacts language or cognitive abilities, and (ii) primary exposure to Dutch as a first language for six-and-a-half or more days per week (i.e. typical monolingual input). Additionally, among tested children, we excluded those with non-responses on 50% or more of the critical trials on a given task from the analyses that involved scores from those tasks.

### 2.2. Materials and procedure

Participants completed three sessions separated by at least 1 day and a maximum of one month between the first and last sessions. The procedures for each session are described below.

#### 2.2.1. Pre-test session

In the first session, participants completed an offline sentence comprehension task (‘pre-test’), the EF and short-term memory tasks and a portion of the standardized language assessment.

In the offline comprehension task, children heard a spoken sentence and were asked to pick the matching picture out of four options. Out of 24 total trials, eight contained active (e.g. *Het paard kietelt de aap/The horse tickles (is tickling) the monkey*), eight passive (e.g. *Het schaap wordt gekieteld door de muis/The sheep is tickled (is being tickled) by the mouse*) and eight intransitive (e.g. *De beer huilt/The bear cries (is crying*)) sentences.^1^ The accompanying pictures for active and passive trials comprised the target corresponding to the sentence (e.g. picture of horse tickling monkey), the reversal corresponding to the syntactic reversal of the sentence (e.g. monkey tickling horse), a distractor where the event participants and roles were the same as the target but the action was different (e.g. horse painting on the monkey) and a distractor where the action was the same but the event participants were different (e.g. goat tickling cow). Thus, choosing the matching target picture required accurate syntactic comprehension and could not be accomplished by simply understanding the individual words. The pictures for intransitive trials similarly comprised the target, a distractor with a different action and a distractor with different event participants. Since syntactic reversal is not possible for intransitive, the fourth picture depicted a different animal and a different action from the target. Four transitive verbs (*kietelen/tickle, verven/paint on, trekken/pull* and *schoppen/kick*) appeared four times each, twice in active and twice in passive structures. Four intransitive verbs (*huilen/cry, zwaaien/wave, slapen/sleep* and *zwemmen/swim*) appeared twice each. Eight animals (*paard/horse, beer/bear, koe/cow, aap/monkey, schaap/sheep, geit/goat, leeuw/lion* and *muis/mouse*) appeared five times, once each as the agent and the patient in active and passive sentences and once as the agent in intransitive sentences. The full set of materials is shown in the appendix.

Children were familiarized with the animals and their labels prior to testing. The target picture appeared equally often in the four quadrants. The order of the sentences was pseudorandom such that no more than two consecutive sentences had the same structure. The task was administered interactively with the experimenter speaking the sentences aloud and the children choosing the matching picture from a printed booklet. This made the pre-test (and the corresponding post-test) intentionally different from the exposure task (see more below). We computed the mean accuracy on passive trials as follows: number of passive trials with a correct response/number of passive trials with a picture choice response. For trials with multiple responses, we coded the final response from the participant.

The Day/Night task is a standard measure of EF, specifically inhibition or interference control [34,35]. Children were asked to say ‘night’ (*nacht*) when they saw a picture of the sun and ‘day’ (*dag*) when they saw a picture of the moon. Thus, the task required controlling interference from a prepotent or dominant response (e.g. saying ‘sun’ in response to a picture of the sun) and selecting and producing an alternative response. After practice to ensure that participants understood the task, 16 test trials were administered where children’s verbal responses to pictures were recorded on a scoresheet. The order of trials was pseudorandomized such that there were no more than three of the same picture in a row. Each child’s Day/Night score was calculated as follows: number of trials with a correct response/number of trials with a label response. For trials with multiple responses, we coded the initial response because an incorrect initial response followed by a repair could indicate a failure to inhibit, which is relevant to our hypotheses.

The Go/No-Go task is widely used to measure motor inhibition [38]. Children were asked to press a key (‘k’ marked with a green sticker) to catch a fish when they saw it and withhold any response when they saw a shark instead. Similar to the Day/Night task, this required inhibiting a response. However, there was no additional requirement for selecting an alternative response. Trials were administered using E-prime. After practice, children received 60 test trials—comprised of five pairs (10 trials) where a single Go trial was followed by a No-Go trial, five quartets (20 trials) where three successive Go trials were followed by a No-Go trial and five sextets (30 trials) where five successive Go trials were followed by a No-Go trial. Order was randomized such that there were no consecutive pairs, quartets or sextets. Inhibition of the motor response could get harder after a higher number of preceding Go trials. For the present purposes, however, we computed scores collapsed across all trials. On each trial, the child saw a picture of a fish or a shark for 2000 ms or until a response was recorded. No feedback was provided. The inter-stimulus interval was 1000 ms. The No-Go accuracy was computed as follows: number of No-Go trials with a correct response (i.e. no response)/number of No-Go trials. Performance on this task can also be characterized using d′. Because d′ is highly correlated with No-Go accuracy in the age groups tested [38], we used the latter, simpler measure.

The Flanker/Reverse Flanker task [37] is a complex EF task that involves working memory, selective attention, cognitive flexibility and set-shifting. In the first block (Flanker), children saw blue fish and were asked to press the arrow direction (left or right) matching the direction of the middle fish (17 trials). This required ignoring distraction from the direction of the Flanker fish surrounding the middle fish. In the second block (Reverse Flanker), children saw pink fish and were asked to press the arrow direction corresponding to the Flanker rather than the middle fish (17 trials). This required ignoring distraction from the middle fish and suppressing the rule used in the first block. In the third block (Mixed), the two tasks were pseudorandomly intermixed, requiring children to flexibly shift between the two rules (65 trials). In all blocks, congruent trials contained five fish that were pointing in the same direction. On incongruent trials, the middle fish and the surrounding four fish were pointing in opposite directions.

Each block included practice to make sure that children understood the task(s). The left arrow and right arrow stickers were pasted on the ‘z’ and ‘/’ keys (on opposite ends of the keyboard). Two sheets of paper with a hand drawn on them were placed near these keys. Children were asked to return their hands to these locations at the end of each trial. Trials were administered using E-prime. On each trial, children saw a blank screen (500 ms) and then a picture with the fish until a response was recorded, followed by a 1000 ms inter-trial interval. Following Qi *et al*. [15], we used accuracy on incongruent trials during the third block as a measure of inhibition in the context of task switching. Each child’s score was computed as follows: number of trials with a correct response/number of eligible trials (see below). Trials with responses faster than 200 ms were excluded from the calculation because they are likely to be anticipatory and unlikely to reflect the application of a rule after processing the visual stimulus [37].

The word span task was used to measure verbal short-term memory [45]. The experimenter spoke aloud strings of words at the rate of one word per second. Children were asked to listen to each entire string and repeat the words in the same order. After practice, they received six trials each of length of two to six words (30 trials total). One hundred and twenty single-syllable Dutch words known commonly to young children (Lexilijst Nederlands [46]) each appeared once in the experiment. A child had to get at least four out of six trials correct at a given length to move on to the next length (i.e. testing stopped whenever the child got three trials wrong for a length). If a child got the first four trials right in length, the next two were skipped, and they received credit for all six trials [45]. Each child’s word span score was calculated as: number of correct trials (the right words in the right order. Maximum score = 30). For trials with multiple responses, we coded the final response from the participant.

#### 2.2.2. Exposure session

In the second session, children completed an online sentence processing task and a portion of the standardized language assessment.

**Figure 1.**
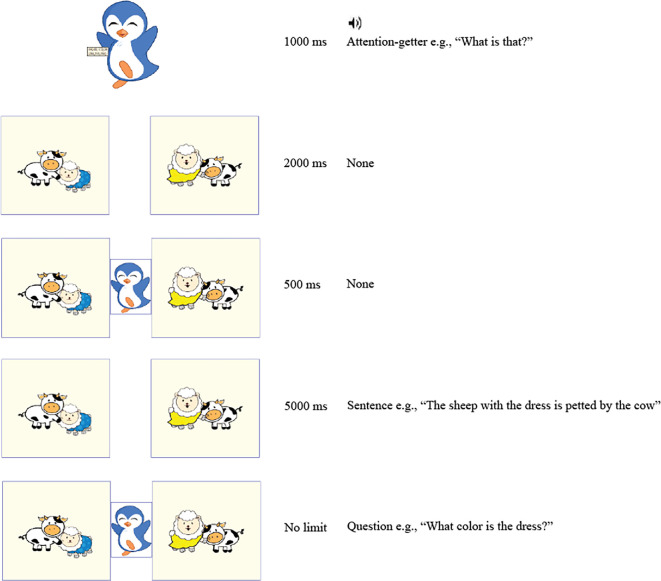
Schematic of the trial procedure during the exposure session.

In the online sentence processing task, they heard active and passive sentences (e.g. *Het schaap met de jurk wordt geaaid door de koe*/*The sheep with the dress is petted by the cow*) and saw two pictures side by side on a screen (see appendix for the full list of sentences and figure 1, for example, pictures). One picture showed the action corresponding to the sentence (e.g. cow petting sheep), and the other showed the same action with the agent and the patient reversed (e.g. sheep petting cow). We tracked and analysed children’s eye movements to the two pictures time-locked to the relevant parts of the sentence. The analyses were designed to reveal whether children had a first-NP-is-agent bias that caused them to initially interpret passive structures as if they were active (looking at the reversal picture), and, if so, whether they revised that initial misinterpretation to arrive eventually at the correct interpretation (looking at the target picture). A cartoon penguin appeared periodically during the experiment to provide non-specific encouragement and ask questions. Each sentence contained a modifier phrase after the first noun (e.g. *with the dress*) in order to lengthen the period of syntactic ambiguity. The animal corresponding to the first noun was wearing the clothing item (e.g. dress) mentioned in both pictures, but the colour of the item was different in the two cases (blue and yellow always, with the correct answer counterbalanced across trials). At the end of each trial, the penguin asked the children to name the colour (e.g. *Welke kleur heeft de jurk?*/*What colour is the dress?*). Children’s verbal answers served as additional offline measures to complement the online eye movement measures. We used the answers as indirect indicators of children’s final picture choice instead of asking children directly about who did what to whom (e.g. whether the cow petted the sheep or vice versa) because we did not want to draw explicit attention to the manipulations or inadvertently prime syntactic structure via the form of the question. We opted for a verbal response over pointing because the latter could potentially cause track loss during eye-tracking. Additionally, having distinct response modalities during pre-test/post-test (pointing) and exposure (verbal) provided a stronger test of whether children were acquiring linguistic representations and not just learning to do a task.

The trial procedure is shown schematically in figure 1. Each trial began with the penguin appearing in the middle and saying something to draw children’s attention and/or provide encouragement (e.g. *Wat is dat?/What is that?, Fantastisch/Fantastic*. 1000 ms). Subsequently, the two pictures were shown so children could visually process them before the auditory input (2000 ms). The penguin then reappeared briefly in the middle to draw children’s eyes to the centre (500 ms) before the sentence was played (5000 ms). After the end of the sentence, the penguin appeared and asked a question about the colour of the clothing item while the two pictures remained on the screen. The experimenter recorded the children’s answers and proceeded to the next trial by pressing a key.

Children were familiarized with the animals, actions and the task before the experiment began. First, they saw printed pictures of the animals used in the critical stimuli and heard the corresponding nouns. Subsequently, they saw printed pictures of the actions and heard active sentences describing the actions. The sentences used during action familiarization contained different nouns (i.e. animals in the pictures) from the test, so there was no overlap of sentences or scenarios with the actual experiment. Finally, children received three practice trials in front of the eyetracker, where they heard an active sentence containing different nouns and verbs than the experiment, saw two pictures on the screen and were asked to answer the offline comprehension question (as described above). If children appeared unsure or gave the wrong answer on a practice trial, we repeated the sentence and asked them to identify the matching picture and then identify the colour of the clothing item in that picture. Subsequently, we reaffirmed a correct answer or corrected a wrong answer before moving on to the next practice trial. During the experiment, children received 48 intermixed active and passive trials (24 of each). The order was pseudorandomized such that the same structure was heard a maximum of two times in a row. The correct target picture appeared on the left or right side of the screen an equal number of times (24 each) with the additional constraint that the correct side (e.g. left) repeated a maximum of two times in a row. The side was also counterbalanced within each structure (12 each of active/passive combined with left/right side). Within a picture, the relative location of the agent and the patient was also counterbalanced. In half the trials, the agent was located to the left of the patient (in both pictures). The correct answer to the offline question was *blue* or *yellow* an equal number of times, with the additional constraint that the same correct answer repeated a maximum of three times in a row. No feedback was provided during the critical trials. The target picture for each trial was counterbalanced across two lists (e.g. list 1: *The sheep with the dress is petted by the cow*; list 2: *The sheep with the dress pets the cow*). For each list, the 48 trials were split into three blocks of 16, which appeared in all six possible permutations of orders. Each child was assigned to one of the 12 possible stimulus presentation conditions.

The animals in the pictures were the same as during pre-test and post-test (eight animals each appearing 12 times balanced between active and passive structures and agent and patient roles). The verbs were different from pre- and post-test because we intended to evaluate whether exposure to passive sentences resulted in learning about the abstract passive structure rather than verb-specific associations. Six transitive verbs (*wassen/wash, duwen/push, dragen/carry, aaien/pet, vangen/catch* and *kammen/comb*) appeared a total of eight times, equally often in active and passive sentences.

For eye-tracking, children sat in a chair in front of the tracker (Eyelink 1000, 890 nm illuminator, 25 cm lens) with a target sticker on their forehead. Accompanying adults sat behind the children and wore headphones listening to masking music. Videos were displayed on a monitor (55 cm, 1920 × 1080 pixels), and audio was played over speakers located to the left and right behind the screen. Tall room dividers prevented children from seeing the experimenter, who controlled stimulus presentation and recorded children’s verbal answers. The session was video recorded using OBS Studio (21.1.0) and a webcam. Participants were calibrated before each block of 16 sentences. The left eye was tracked.

#### 2.2.3. Post-test session

The final post-test session closely resembled the pre-test session in that participants completed an offline comprehension test, the EF and short-term memory tasks and a portion of the standardized language assessment.

For offline comprehension, the procedure was the same as for pre-test. The order of trials was pseudorandom and subject to the same constraints. We counterbalanced the materials such that the reversal pictures during the critical transitive trials at pre-test were the target pictures at post-test and vice versa (e.g. pre-test: target = horse tickles monkey, reversal = monkey tickles horse. Post-test: target = monkey tickles horse, reversal = horse tickles monkey). Thus, no sentences or target pictures were repeated between the two sessions. The distractor pictures and the pictures for intransitive trials followed the same logic as before. Half the children got set 1 during pre-test and set 2 during post-test and the other half got the reverse (see appendix).

The tasks for measuring EFs and short-term memory were the same as during pre-test. These tasks were repeated so we could assess test–retest reliability.

### 2.3. Dependent measures and analyses

The analyses focused on three hypotheses arising from Pr1 and Pr2, namely that EF (as defined here) facilitates the revision of initial misinterpretations of passive structures, leading to lasting changes in children’s syntactic knowledge. Below, we describe each hypothesis and analysis in turn. See table 1 for a summary of the stated and alternative hypotheses and table 2 for analysis decision parameters.

**Table 1.** Study design. EF = executive function, DV = dependent variable, IV = independent variable.

question	stated hypothesis	alternative hypothesis	analysis plan	interpretation given different outcomes
does EF improve the efficiency of revising the online interpretation of passives?	**hypothesis 1.** negative correlation between EF score and latency to switch from the wrong (active) to the right (passive) picture after a sentence is disambiguated.	EF will not correlate with latency to switch to the correct picture if incremental revision is guided by the syntactic parser and not by non-sentential EF.	multiple regression DV = mean log-transformed latency to switch from wrong to right picture. Critical IV = aggregate EF score. Control IVs = pre-test, receptive language and short-term memory scores plus mean log-transformed latency from active trials. ^2^^,^ ^3^	if the regression yields a negative correlation between aggregate EF and the DV, we will claim support for hypothesis 1. if there is no significant correlation, we will conclude that there is no evidence for the stated hypothesis. we do not anticipate positive correlations (better EF associated with poorer online processing).
does EF improve the accuracy of revising the online interpretation of passives?	**hypothesis 2**. positive correlation between EF score and accuracy of switching from the wrong (active) to the right (passive) picture after a sentence is disambiguated.	EF will not correlate with accuracy in switching to the correct picture if incremental revision is guided by the syntactic parser and not by non-sentential EF.	multiple regression DV = revision accuracy score. Critical IV = aggregate EF score. Control IVs = pre-test, receptive language and short-term memory scores.	if the regression yields a positive correlation between aggregate EF and the DV, we will claim support for hypothesis 2. if there is no significant correlation, we will conclude that there is no evidence for the stated hypothesis. as before, we do not expect correlations in the opposite direction.
do better online revision and EF lead to improved acquisition of passives?	**hypothesis 3**. positive correlation between online revision score and improvement in passive comprehension from pre- to post-test. **hypothesis 3a**. positive correlation between EF and improvement in passive comprehension from pre- to post-test.	online revision score will not correlate with improvement in passive comprehension if long-term syntactic acquisition is guided by offline rather than online skills (e.g. mapping syntactic templates to meaning, pragmatic reasoning). EF will not correlate with improvement in passive comprehension if long-term syntactic acquisition is guided by offline linguistic skills such as the above or by other cognitive abilities like analogical reasoning that are not subsumed under the definition of EF used here.	Multiple regression DV = post-test score minus pre-test score. Critical IV = revision accuracy score. Control IVs = pre-test, receptive language and short-term memory scores. Same as (1) but with the aggregate EF score as the critical IV.	if the regression yields a positive correlation between the DV and both revision accuracy and aggregate EF, we will conclude that the acquisition of passives is supported by online revision, which is in turn facilitated by EF. if the regression yields a positive correlation between the DV and revision accuracy but not aggregate EF, we will conclude that acquisition is related to online revision abilities but that there is no evidence that the relevant mechanism is EF. if the regression yields a positive correlation between the DV and aggregate EF but not revision accuracy, we will conclude that EF facilitates the acquisition of passives in some other way than by facilitating online revision (e.g. offline decisions). if the regression yields no correlation between the DV and both revision accuracy and EF, we will conclude that there is no support for a link between online processing and long-term syntactic acquisition and for EF’s role in long-term syntactic acquisition. as before, we do not expect correlations in the opposite direction.

**Table 2.** Analysis decision parameters.

hypothesis	fixed parameters	what can change
1	DV = mean switch latency from log-transformed reaction times critical IV = aggregated EF *z* score	the full multiple-regression model is intended to have up to four control IVs (pre-test, receptive language and short-term memory scores for all models, and active sentence latency for hypothesis 1 only). To avoid collinearity, any control IV that is correlated with the critical IV (*r* > 0.8) will be removed from the model, and the interpretation will be modified to state that we cannot separate the contributions of the correlated variables. If two control IVs are correlated with one another (*r* > 0.8), we will compute a composite *z* score from the two correlated variables and use that in the model. Interpretation of any associated results will then be modified accordingly.
2	DV = mean accuracy of switching critical IV = aggregated EF *z* score	
3	DV = post-test minus pre-test analysis 1: critical IV = revision accuracy score analysis 2: critical IV = aggregated EF *z* score	

We based our sample size on a rule of thumb for 80% power to detect a medium effect size (*R*^2^ = 0.07) and test the significance of individual predictors [47]. We chose a multiple of 12 (for 12 lists) above this recommendation (*n* ≥ 104 + *k*, *k* = number of predictors = maximum 6). For comparison, the two prior studies that have reported a correlation between EF and the revision of sentence interpretation used sample sizes of 38 and 40 [15,16]. Our study is powered to detect a much smaller effect than that found by Woodard *et al*. [16], who also used multiple regression (table 4; adjusted *R*^2^ = 0.42).

Interpretation of individual difference effects is contingent on whether those differences are stable and can be reliably measured [48]. For EF, previous evidence suggests that differences between individuals persist over development [49,50]. To evaluate reliability, we calculated test–retest reliability across pre- and post-test measurements using an aggregate score, which should be less susceptible to task-specific noise than individual scores.

For online processing, many prior studies have demonstrated the sensitivity and utility of eye movement measures for testing hypotheses about language processing and acquisition (e.g. [51–55]). Individual differences in linguistic and cognitive abilities detectably manifest in eye movement differences during sentence processing in young children, adolescents and adults [51,53–55]. Thus, eye movements can reliably reflect individual differences that influence language processing, as we expected for hypotheses 1 and 2 below. Further, individual differences in eye movements are reliable enough to explain variance in longer-term language acquisition, as we expected for hypothesis 3 below [52]. In sum, the ecological validity of using eye movement measures to study language processing is well-established.

Studies conducted in two of the authors’ labs have independently found that latencies to switch from the incorrect to the correct picture during language processing can be measured reliably in young children [56,57]. Donnelly & Kidd [56] reported that switch latency at 18 months correlated with that at 21 and 24 months. Peter *et al*. [57] found significant correlations between 19 months and 25 and 31 months. In the present study, practical constraints on the study duration meant that we could not include multiple eye-tracking sessions and examine test–retest reliability. Instead, we assessed within-session Spearman–Brown split-half reliability. Based on prior work in our labs, we expected reliable measurements but also considered how the interpretation of the results would need to be modified in the event of insufficient reliability. Were this to be the case, hypotheses 1, 2 and 3 would require future studies to determine how reliability could be improved. However, hypothesis 3a can still be evaluated. Thus, the present study will have theoretical impact—under all contingencies—because it will shed light on whether syntactic development can be influenced by a specific, non-syntactic EF mechanism (i.e. inhibition). It will also make an empirical contribution on using eye movement measures appropriately in language acquisition because little is known about the reliability of these measures for indexing individual differences in syntactic (cf. lexical) processing.

Language-mediated eye movements, like the ones measured here, are endogenously rather than exogenously driven. As such, they are expected to show protracted development during childhood and sensitivity to differences in EF [58]. However, eye movement differences due to lower level perceptual components cannot be ruled out [53,59]. Therefore, to isolate the critical component of interest, namely revision from misinterpretation, we included mean saccade latencies during the active sentence trials as a covariate in the relevant models (see table 1).

The hypotheses were tested using four different multiple-regression models. For each model, we report which results were significant with Bonferroni correction (*p* < 0.0125) and without correction (*p* < 0.05) and provide effect sizes to aid appropriate interpretation.

### 2.4. Hypothesis 1: Executive function and the efficiency of revising an interpretation during online processing

Our first hypothesis was that better EF would result in higher efficiency in revising an initial misinterpretation. Thus, latency to switch from looking at the incorrect picture (corresponding to an active interpretation) to the correct picture (corresponding to a passive interpretation) during online processing (in the exposure session) should be correlated with individual differences in EF.

Only passive trials were analysed in this and all subsequent analyses (active trials were not relevant to the hypothesis). Here and below, all time intervals for analyses were shifted by 200 ms to account for the time taken to process incoming linguistic information and programme a saccade, per standard practice in the field. Because we were interested specifically in revision, we only examined trials where the child was looking at the active interpretation picture prior to syntactic disambiguation, namely at the onset of the auxiliary verb (*wordt/is*, plus 200 ms). The analysis interval ended at the onset of the second NP (plus 200 ms) because subsequent looks are likely to be guided by trying to identify the referent of the noun and not syntax alone. Latency to switch was computed as the time from the beginning of the analysis interval to when the child initiated a saccade to the passive interpretation picture. Trials, where the child had not switched by the end of the analysis interval, were coded as the maximum interval duration. Trials, where the child was not looking at the screen for at least 100 ms during the critical window, were excluded because they likely involved general inattention and could lead to inaccurate latency estimates.^4^

For each participant, we computed the average switch latency from log-transformed reaction times. Multiple linear regression was used to test whether the aggregated EF score predicted variance in switch latency. Pre-test, receptive language and short-term memory scores were added as control regressors. Additionally, to control for eye movement differences unrelated to revision, we included the average saccade latency from log-transformed reaction times during active sentence trials. We included all movements following the onset of the verb to the end of the sentence on trials where the child was looking at the screen for at least 100 ms during the analysis window (as above). To avoid collinearity, here and below, we checked for significant correlations between the regressors and omitted or collapsed across control variables as needed (see table 2).

This hypothesis and the one below propose an association between EF and revision from initial misinterpretation, *when it happens*. We do not make any assumptions about the likelihood of the initial misinterpretation itself. Children may or may not differ in their first-NP-is-agent bias (evidence for this bias in adults and in speakers of different languages suggests that it is near universal). Importantly, we restricted the analyses to only the trials where children were looking at the active interpretation picture prior to disambiguation. Thus, we compared like to like regardless of any such uncontrolled differences between children. We also included pre-test and receptive language scores as covariates in all the analyses to help capture alternative sources of variability in sentence comprehension.

### 2.5. Hypothesis 2: Executive function and the accuracy of revising an interpretation during online processing

In addition to, or instead of, any effects on efficiency or the speed of revision, better EF could also lead to higher accuracy in revising initial misinterpretations. Thus, for this analysis, we looked at a dichotomous ‘switched or not’ measure. As before, only trials where the child was looking at the active interpretation picture at the onset of the auxiliary verb (plus 200 ms) were examined. Within this subset, cases where the child switched to the passive interpretation picture at some point prior to the second NP onset (plus 200 ms) were coded as 1 and the others were coded as 0.

The dependent variable for this analysis was a mean revision accuracy score, computed as the average accuracy for each participant. As before, we used multiple regression to test whether individual differences in the aggregated EF score explained variance in the dependent variable (above and beyond the contributions of the control variables). We assessed the distribution of the dependent variable and used the untransformed variable if the distribution was normal (as was found in Woodard *et al*. [16], figure A2) or transformed the variable if normality was violated.

### 2.6. Hypothesis 3: Acquisition of the passive structure and its relationship to revision during online processing and executive function

Our third hypothesis was that better revision ability during online processing would be associated with better acquisition of the passive structure because it assists in the accurate interpretation of passive sentences heard in the language input. The dependent variable for this analysis was (post-test score minus pre-test score). The critical predictor was the revision accuracy score from the exposure session. Pre-test, receptive language and short-term memory scores were included as control regressors. Multiple regression was used to test whether revision accuracy predicted variance in how much passive comprehension scores improved from pre- to post-test.

It is worth clarifying that per the hypothesis, EF was expected to impact the acquisition of the passive structure via its influence on how accurately passive sentences in the input are processed. This is why we used revision accuracy and not EF as the predictor in the main analysis. However, an alternative hypothesis could be that EF predicts the acquisition of passives independently of online revision (e.g. via helping offline comprehension). To test for this possibility, we also conducted a secondary analysis using the aggregated EF score instead of revision accuracy as a predictor. This will allow us to determine if and how EF impacts not just the online processing of passive structures but also their acquisition over time.

## 3. Results

This study was conducted using the procedures specified in the approved stage 1 protocol here: https://osf.io/yqvbf/?view_only=1a865e3a65104be686f563fac5d5957f. All data and scripts can be found here: https://osf.io/atj3v/?view_only=7fd6da72b89d4471b7d5a36479586cc1.

One hundred and forty-nine children were tested. Twenty-nine were excluded for the following reasons: did not complete all three sessions or all three EF tasks during pre-test (*n* = 20), refused to do a task, pressed buttons randomly or parent intervened (*n* = 8) and had a developmental disorder (*n* = 1). The remaining 120 participants (67 F, 53 M. Mean age = 51.5 months) comprised the initial dataset for analysis. For each hypothesis, there were additional exclusions for other reasons (e.g. eye-tracking data was not usable). Below, we specify the number of participants included in each analysis along with the associated details.

### 3.1. Executive function measures

Each child (*n* = 120) completed the three EF tasks—Day/Night, Go-No-Go, Flanker/Reverse Flanker—during the pre-test session. Descriptively, the Flanker/Reverse Flanker task showed the lowest mean accuracy (67.4%) and wide individual variability (s.d. = 21.1). The Day/Night task showed slightly higher mean accuracy (73.8%) and also wide variability (s.d. = 26.0). The Go-No-Go task appeared to be the easiest for the children (mean = 88.3%, s.d. = 18.2). For the post-test session (*n* = 114, six children did not complete one or more tasks), the corresponding scores were: Flanker/Reverse Flanker (mean = 73.3%, s.d. = 19.6); Day/Night (mean = 79.8%, s.d. = 19.6) and Go-No-Go (mean = 89.3%, s.d. = 13.2).

We computed *z* scores for each task in the pre- and post-test sessions and evaluated test–retest reliability using the intraclass correlation coefficient (ICC). ICCs for single and average measures were as follows—Flanker/Reverse Flanker (single = 0.51, average = 0.68); Day/Night (single = 0.43, average = 0.60) and Go-No-Go (single = 0.07, average = 0.14). Go-No-Go showed poor reliability for detecting individual differences. Flanker/Reverse Flanker had the best reliability. In all cases, averaging across sessions was better than using scores from a single session. Aggregating across all three tasks, the ICC was 0.48 (single) and 0.65 (average). Because Go-No-Go showed poor reliability, we also aggregated across just Flanker/Reverse Flanker and Day/Night. ICC for this two-task aggregate was 0.61 (single) and 0.76 (average).

Pairwise correlations between the three tasks revealed that the average Flanker/Reverse Flanker score was significantly correlated with both the average Day/Night (*r* = 0.35, *p* < 0.001) and average Go-No-Go (*r* = 0.32, *p* < 0.001) scores. However, average Day/Night and average Go-No-Go did not significantly correlate with one another (*r* = 0.08, *p* = 0.42).

Overall, averaging across sessions and aggregating across tasks improved reliability. Below, we use the average aggregate across the three tasks in the models, as proposed during pre-registration. In electronic supplementary materials, we additionally report results using the average aggregate across Flanker/Reverse Flanker and Day/Night because this score had the highest ICC out of all the measures.

### 3.2. Eye-tracking measures

In the exposure session, for each child, we computed the time it took to switch from the wrong picture to the correct picture for passive sentences within the interval of interest (i.e. from the onset of *wordt [is]* (plus 200 ms) to the onset of *de/het [the]* (plus 200 ms)). Trials where the child was looking at the screen for less than 100 ms during this interval were excluded (13.1%). Switch latency was computed only for trials where a child was looking at the wrong picture (i.e. the one that matches an active sentence structure) at the beginning of the interval. This criterion eliminated a large percentage of the trials (57.6%).

For the eligible trials, the switch latency was the time it took to switch, or the full interval duration (in cases where children did not switch by the end of the interval). Some participants had very few eligible trials, hindering the computation of split-half reliability (‘splithalf’ function in R). We computed split-half reliability using a minimal threshold (≥4 eligible trials) for which computation was possible (*n* = 80). Using 5000 random splits, the Spearman–Brown corrected reliability estimate was 0.18 (95% confidence interval (CI) (−0.1, 0.42)).

For the eligible trials, revision accuracy was set to 1 if a participant switched from the wrong to the right picture within the interval, and 0 if not. Using the same threshold (≥4 eligible trials) and parameters as above (*n* = 80), the Spearman–Brown corrected reliability estimate was 0.43 (95% CI (0.26, 0.59)).

Overall, the small number of trials that were eligible per participant according to the criteria established during pre-registration (mean = 6.6, range = 1–19) might have impacted the reliability of the eye movement variables. Switch latency (the DV for hypothesis 1) showed poor reliability. By comparison, the dichotomous revision accuracy measure (the DV for hypothesis 2) showed fair reliability.

### 3.3. Collinearity between predictors

Table 3 shows the matrix of correlations between the IVs for the different hypotheses. None of the correlations were above the pre-registered threshold (*r* > 0.8). However, receptive language was significantly correlated with the critical IVs as well as other control IVs at Bonferroni-corrected *p* < 0.005. The other control IVs (pre-test passive and short-term memory score) were not correlated with each other or other variables. Given this pattern of correlations, we report below two kinds of analyses: (1) the pre-registered analyses with all control IVs included in the models, and (2) exploratory analyses that exclude receptive language from the models and examine potential mediation of any EF effects by this control IV.

**Table 3.** Correlation matrix for predictors.

hypothesis	correlation type	pre-test passive	receptive language	short-term memory
#1, 2, 3A critical IV: average aggregate EF score	between critical IV and control IV	0.18	0.56**	0.23^a^
#3 critical IV: revision accuracy	between critical IV and control IV	0.11	0.34**	0.16
all	between control IVs	receptive language correlated with both pre-test passive (0.30**) and Short-term memory (0.29**), which were not correlated with each other (−0.03).		

^a^ Indicates *p* < 0.05, bold and ** indicates *p* < 0.005. Eye movement latency during active trials, which was a control IV for hypothesis 1 only, is not shown. It did not correlate with any of the variables (all *p*’s > 0.3).

### 3.4. Clarifications about the short-term memory scores

In the word span task, the intended administration required testing to stop whenever the child got three trials wrong for a length. If a child got the first four trials within a length category (e.g. sequence of three words) correct, they were supposed to receive automatic credit for all six trials (i.e. the next two trials were supposed to be skipped). We noticed after data collection that these rules were applied inconsistently across the sample, leading to some children receiving fewer trials and others receiving more trials than they should have. Therefore, we adapted the scoring procedure to compute the short-term memory score as: number of correct trials/number of trials administered. Although this deviates from the normative scoring procedure, the significant correlation between the short-term memory score and children’s receptive language (see above) suggests that the modified procedure was able to capture individual differences that are relevant to language acquisition.

### 3.5. Hypotheses: Pre-registered analyses

In this section, we discuss the results from the models specified during pre-registration. In the section below, we discuss other exploratory analyses. Hypotheses 1 and 2 tested whether online processing measures were predicted by children’s EF. Hypothesis 1 tested the effect of EF on the (log-transformed) latency to switch from the wrong to the right interpretation during online processing, with receptive language, pre-test passive score, short-term memory and latency during active trials included as control variables (*n* = 96 after excluding participants with missing eye-tracking or EF data). The overall model was not significant (*p* = 0.27), and there were no significant individual effects (though note that for receptive language *p* = 0.05; table 4). Hypothesis 2 tested the effect of EF on revision accuracy during online processing, with receptive language, pre-test passive score and short-term memory included as control variables (*n* = 96, as above). This time, the overall model was significant (*p* = 0.008) but once again, there were no significant individual effects (though note again that for receptive language *p* = 0.05; table 4).

**Table 4.** Results from the pre-registered model for hypothesis 1 (predictors of latency to switch) and hypothesis 2 (predictors of revision accuracy) during online processing.

hypothesis 1: latency to switch multiple *R*^2^ = 0.07, adjusted *R*^2^ = 0.02. *F*(5,90) = 1.31, *p* = 0.27					
	estimate	SE	*t*	*p*	partial eta^2^
(intercept)	6.85	0.28	24.28	<0.001	
average aggregate EF score	−0.02	0.05	−0.42	0.67	0.02
receptive language	−0.005	0.003	−1.95	0.05	0.03
pre-test passive	0.001	0.001	1.18	0.24	0.01
short-term memory	0.15	0.22	0.67	0.50	<0.01
(log) active trials latency	−0.002	0.06	−0.03	0.98	<0.01

hypothesis 2: revision accuracy during online processing multiple *R*^2^ = 0.14, adjusted *R*^2^ = 0.10. *F*(4,91) = 3.64, *p* = 0.008					
	estimate	SE	*t*	*p*	partial eta^2^
(intercept)	−0.08	0.22	−0.38	0.70	
average aggregate EF score	0.06	0.05	1.21	0.23	0.09
receptive language	0.006	0.002	1.95	0.05	0.05
pre-test passive	0.0002	0.001	0.17	0.87	<0.01
short-term memory	0.14	0.23	0.62	0.54	<0.01

**Figure 2.**
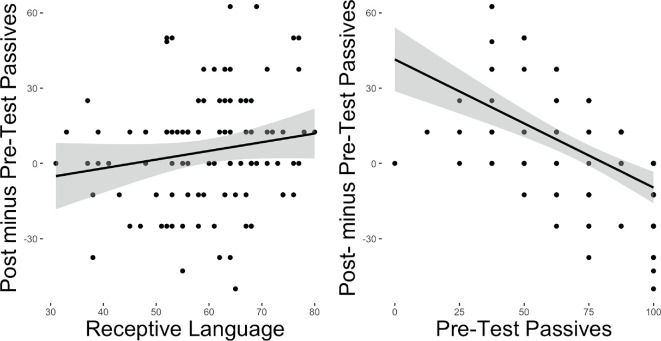
Correlation between pre-test to post-test improvement and receptive language (left) and pre-test passive score (right).

Hypotheses 3 and 3a tested whether improvement in passive comprehension accuracy from pre-test to post-test was predicted by revision accuracy (3) and EF (3a), respectively. In both cases, the model included the control variables receptive language, pre-test passive score and short-term memory. For hypothesis 3 (*n* = 96, as above), there were significant effects of receptive language and pre-test scores (table 5). Children with better receptive language showed a larger improvement from pre-test to post-test (figure 2: left panel). Those with higher pre-test scores showed a smaller increase, potentially because those with higher pre-test scores had more limited room for improvement (figure 2: right panel). Revision accuracy (the critical IV) did not significantly predict pre- to post-test improvement. For hypothesis 3a (*n* = 114, no eye-tracking measures in the model), there were significant effects of receptive language and pre-test passive score (similar to hypothesis 3) but not EF (table 5).

**Table 5.** Results from the pre-registered models for predictors of passive comprehension improvement from pre- to post-test: hypothesis 3 (revision accuracy was predictor of interest) and hypothesis 3a (EF was predictor of interest).

hypothesis 3: pre- to post-improvement (critical IV = revision accuracy) multiple *R*^2^ = 0.42, adjusted *R*^2^ = 0.39. *F*(4,91) = 16.17, *p* < 0.001					
	estimate	SE	*t*	*p*	partial eta^2^
(intercept)	−11.39	14.93	−0.76	0.45	
revision accuracy	−10.78	7.99	−1.35	0.18	<0.01
**receptive language**	**0.76**	**0.20**	**3.86**	**<0.001**	**0.06**
**pre-test passive**	**−0.60**	**0.08**	**−7.30**	**<0.001**	**0.38**
short-term memory	23.82	17.48	1.36	0.18	0.02

hypothesis 3a: pre- to post-improvement (critical IV = EF) multiple *R*^2^ = 0.48, adjusted *R*^2^ = 0.46. *F*(4,109) = 25.45, *p* < 0.001					
	estimate	SE	*t*	*p*	partial eta^2^
(intercept)	−9.93	15.66	−0.63	0.53	
average aggregate EF score	1.81	3.79	0.48	0.63	0.03
**receptive language**	**0.73**	**0.21**	**3.47**	**<0.001**	**0.01**
**pre-test passive**	**−0.69**	**0.07**	**−9.44**	**<0.001**	**0.47**
short-term memory	26.14	16.00	1.63	0.11	0.02

### 3.6. Exploratory analyses 1: Modified eye movement measures

The eye-tracking measures—switch latency and switch accuracy—did not have high split-half reliability (see above), which could have impacted our ability to detect effects for the hypotheses that involved those measures (hypotheses 1, 2 and 3). We computed these measures based on eye movements only in the interval between the onset of *wordt* and the onset of *de/het* and further, only for those trials where children were looking at the wrong picture at the beginning of the interval. These criteria resulted in cutting the number of trials available for analyses by more than half, which in turn could have led to poor reliability. Therefore, in exploratory analyses, we extended the interval of interest to 2000 ms after the onset of *wordt* and included trials where the children were looking at the wrong picture at any time during the interval (not just at the beginning of the interval). As before, we computed whether children switched from the wrong to the right picture and the latency to make that switch.

The above-mentioned modifications eliminated fewer trials from analysis than before—trials where the child was looking at the screen for less than 100 ms during the interval (11%, cf. 13.1% before) and trials where they did not look at the wrong picture during the interval (27.9%, cf. 57.6% before). The average number of eligible trials per participant increased from 6.6 to 11.8 and the number of participants with ≥4 eligible trials increased from 80 to 96. We conducted split-half analyses using the same procedures as above. The new Spearman–Brown corrected reliability estimates for switch latency and switch accuracy were 0.64 (95% CI (0.52, 0.73)) and 0.69 (95% CI (0.59, 0.77)), respectively. Thus, the modified criteria yielded eye movement measures with substantially improved reliability. Therefore, we used these new measures in all analyses below.

**Figure 3.**
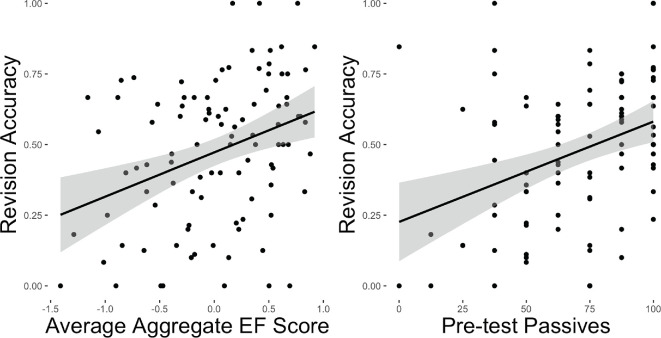
Correlation between revision accuracy (using modified eye-tracking measures) and EF (left) and pre-test passive score (right).

We tested hypotheses 1, 2 and 3 using the new eye-tracking measures. For hypothesis 1, testing the effect of EF, and control variables on latency to switch, there were no significant effects (*n* = 98; table 6, though note that this time the overall model was significant and, for receptive language, *p* = 0.07). For hypothesis 2, testing the effect of EF, and control variables on revision accuracy, there was a significant effect of the EF score and the pre-test passive score (*n* = 99; table 6). Higher EF and pre-test passive scores were correlated with better revision accuracy (figure 3). Children with better EF abilities were more likely to switch from looking at the wrong picture (that matches the active sentence structure) to looking at the correct picture (that matches the passive sentence structure) during online processing. This effect was significant while controlling for receptive language ability, prior performance in passive comprehension and short-term memory. Similarly, children who showed prior evidence for comprehending passives better were better at revision during online processing.

**Table 6.** Results from the models containing modified eye-tracking measures for hypothesis 1 (predictors of latency to switch) and hypothesis 2 (predictors of revision accuracy) during online processing.

**hypothesis 1: latency to switch during online processing** **multiple *R***^**2**^ **= 0.19, adjusted *R***^**2**^ **= 0.15. *F*(5,92) = 4.43, *p* =** 0.001					
	estimate	SE	*t*	*p*	partial eta^2^
(intercept)	7.25	0.31	23.25	<0.001	
average aggregate EF score	−0.09	0.06	−1.58	0.12	0.13
receptive language	−0.006	0.003	−1.84	0.07	0.05
pre-test passive	−0.001	0.001	−1.01	0.32	<0.01
short-term memory	−0.04	0.25	−0.14	0.89	<0.01
(log) active trials latency	0.10	0.06	1.54	0.13	0.03

hypothesis 2: revision accuracy during online processing multiple *R*^2^ = 0.22, adjusted *R*^2^ = 0.18. *F*(4,94) = 6.44, *p* < 0.001					
	estimate	SE	*t*	*p*	partial eta^2^
(intercept)	0.15	0.21	0.73	0.47	
**average aggregate EF score**	**0.11**	**0.05**	**2.11**	**0.04**	**0.13**
receptive language	0.002	0.003	0.88	0.38	0.03
**pre-test passive**	**0.003**	**0.001**	**2.92**	**0.004**	**0.08**
short-term memory	−0.02	0.22	−0.09	0.93	<0.01

For hypothesis 3, testing the effect of online revision on passive comprehension improvement (pre- to post-test), the analysis revealed a significant effect of receptive language and pre-test passive score (*n* = 99; table 7). Similar to the pre-registered analysis, children with better receptive language showed a bigger improvement from pre-test to post-test passive comprehension. Children with higher pre-test scores showed smaller improvements.

**Table 7.** Results from the model containing modified eye-tracking measures for predictors of passive comprehension improvement from pre- to post-test: hypothesis 3 (revision accuracy was predictor of interest).

hypothesis 3: pre- to post-improvement (critical IV = revision accuracy) multiple *R*^2^ = 0.44, adjusted *R*^2^ = 0.42. *F*(4,94) = 18.46, *p* < 0.001					
	estimate	SE	*t*	*p*	partial eta^2^
(intercept)	−10.41	15.14	−0.69	0.49	
revision accuracy	−0.93	8.35	−0.11	0.91	0.03
**receptive language**	**0.75**	**0.20**	**3.81**	**<0.001**	**0.07**
**pre-test passive**	**−0.64**	**0.08**	**−7.68**	**<0.001**	**0.40**
short-term memory	21.68	18.04	1.20	0.23	0.02

### 3.7. Exploratory analyses 2: Mediation analyses

The models thus far for hypotheses 1, 2 and 3a tested whether EF predicts the processing and learning of passives after controlling for other possible predictors, namely, receptive language, pre-test scores and short-term memory. For hypothesis 2, results from the exploratory analyses using the modified eye-tracking measures (though not the pre-registered analysis) revealed that better EF is associated with better revision during online processing, even after controlling for other variables. For hypothesis 1 (pre-registered and exploratory) and hypothesis 3a (pre-registered and exploratory), we did not find EF effects while controlling for other variables. However, although not high enough to be collinear, receptive language did show a moderate correlation with EF (table 3). In addition, receptive language had consistently positive, not insubstantial effects on the dependent variables, though it did not reach significance at *p* < 0.05 in all analyses. These results suggested potentially interesting interactions between EF, receptive language and latency (hypothesis 1) and passive comprehension improvement from pre- to post-test (hypothesis 3a). Therefore, we examined if there was an effect of EF on the dependent variable when receptive language was removed from the models via mediation analysis.

**Figure 4.**
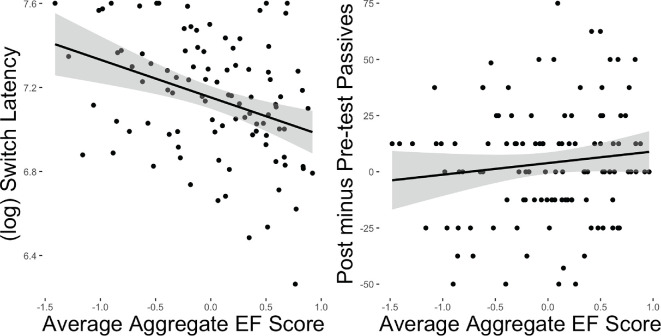
Correlation between EF score and switch latency (with modified eye-tracking measures) for hypothesis 1 and pre-test to post-test improvement for hypothesis 3a.

Mediation analysis can only be attempted if there is a relation between the treatment variable (in our case, EF) and the dependent variable in the absence of the mediator, which we tested first with regression models. There was a significant EF effect on latency to switch (hypothesis 1) and on passive comprehension improvement from pre- to post-test (hypothesis 3a; see table 8) when receptive language was not included in the models. Higher EF scores corresponded with faster switch latencies (figure 4: left panel) and more improvement from pre-test to post-test (figure 4: right panel). Thus, these hypotheses were suitable for mediation analysis.

**Table 8.** Results from the model without the receptive language covariate for hypothesis 1 and hypothesis 3a.

hypothesis 1: latency to switch during online processing multiple *R*^2^ = 0.16, adjusted *R*^2^ = 0.13. *F*(4,93) = 4.57, *p* = 0.002					
	estimate	SE	*t*	*p*	*partial eta* ^*2*^
(intercept)	7.04	0.29	23.98	<0.001	
**average aggregate EF score**	**−0.15**	**0.05**	**−2.86**	**0.005**	**0.13**
pre-test passive	−0.002	0.001	−1.57	0.12	0.02
short-term memory	−0.14	0.25	−0.55	0.58	<0.01
(log) active trials latency	0.09	0.06	1.49	0.14	0.02

hypothesis 3a: pre- to post-improvement (critical IV = EF) multiple *R*^2^ = 0.43, adjusted *R*^2^ = 0.41. *F*(3,110) = 27.19, *p* < 0.001					
	estimate	SE	*t*	*p*	partial eta^2^
(intercept)	19.52	13.80	1.41	0.16	
**average aggregate EF score**	**8.70**	**3.39**	**2.57**	**0.01**	**0.02**
**pre-test passive**	**−0.62**	**0.07**	**−8.39**	**<0.001**	**0.40**
**short-term memory**	**37.17**	**16.45**	**2.26**	**0.03**	**0.04**

We conducted mediation analyses to examine whether receptive language mediated the relationship between EF and the dependent variable (‘mediate’ function in ‘mediation’ package in R). This analysis decomposes the effect of the independent variable on the dependent variable into direct (unmediated) and indirect (mediated) effects, allowing for inferences about the mechanisms underlying the relationship between two variables.

For hypothesis 1, the dependent or outcome variable was switch latency, the ‘treatment’ variable was EF and the ‘mediator’ variable was receptive language. The ‘mediate’ function allows for a binary treatment variable (treatment versus no treatment) or contrasts between levels of a treatment, so we computed quantiles and compared the lowest and highest quartiles of the EF score. The results are shown in table 9 (see model equations in ^5^). The total effect of EF on switch latency is composed of the direct effect (average direct effect) and the mediated effect (average causal mediation effect). As seen in table 9, the total effect was significant but neither the direct nor the mediated effect was significant. Thus, while there is an association between EF and switch latency, we cannot conclude that there is a direct effect of EF on switch latency.

**Table 9.** Mediation analysis for hypothesis 1 (latency to switch) and hypothesis 3a (pre- to post-test improvement).

hypothesis 1: latency to switch during online processing				
	estimate	95% CI lower	95% CI upper	*p*
average causal mediation effect (ACME)	−0.08	−0.18	0.01	0.09
average direct effect (ADE)	−0.13	−0.30	0.07	0.19
**total effect**	**−0.20**	**−0.36**	**−0.04**	**0.02**
proportion mediated	0.37	−0.07	1.93	0.11

hypothesis 3a: pre-test to post-test improvement				
	estimate	95% CI lower	95% CI upper	*p*
**average causal mediation effect**	**9.34**	**3.72**	**16.10**	**0.001**
average direct effect	3.41	−7.65	14.33	0.56
**total effect**	**12.75**	**1.41**	**23.47**	**0.02**
proportion mediated	0.73	0.20	3.38	0.03

For hypothesis 3a, a similar mediation analysis revealed a significant total and a significant mediated effect (table 9; see model equations in ^6^). The direct effect was not significant. Together, this suggests that while EF had an effect on pre- to post-test improvement, this effect was almost all mediated by receptive language.

### 3.8. Exploratory analyses 3: Relationship between comprehension during exposure session and pre- to post-improvement

Did better comprehension of passives during the exposure session lead to more accurate encoding and better learning of passives from experience (pre- to post-test improvement)? Hypothesis 3 above tested this possibility by examining the effect of revision accuracy during online processing on pre- to post-test improvement. Here, we did not find a significant effect either in the model that included (table 7) or in the model that excluded (electronic supplementary material, Table 4) receptive language.

However, we did collect another, offline, measure of comprehension accuracy during the exposure session. Children were asked at the end of each trial to name the colour of a clothing item worn by the animal corresponding to the first noun. The colour was different in the two pictures. Therefore, children’s answers to these questions allowed us to determine their final picture choice (corresponding to an active or passive sentence), without drawing attention to the experimental manipulations of interest. We conducted exploratory analyses to test for an effect of this offline measure on pre- to post-test improvement (no analyses were pre-registered for this predictor). The model containing this critical IV and all control variables, including receptive language, did not reveal a significant effect of offline accuracy, but when receptive language was removed, there was a significant effect (table 10). Higher accuracy for answering comprehension questions was associated with more improvement from pre- to post-test. Next, we conducted a mediation analysis, similar to above, to investigate whether the effect of offline accuracy was mediated by receptive language (table 11; see model equations in ^7^). The total and mediated effects were significant but the direct effect was not, suggesting that offline accuracy was associated with pre- to post-test improvement but this relationship was almost all mediated by receptive language.

**Table 10.** Results for the exploratory analyses of the effect of offline accuracy on pre- to post-improvement.

model with receptive language included as a control variable multiple *R*^2^ = 0.42, adjusted *R*^2^ = 0.40. *F*(4,95) = 17.34, *p* < 0.001					
	estimate	SE	*t*	*P*	partial eta^2^
(intercept)	−15.68	14.80	−1.06	0.29	
offline accuracy	18.57	11.89	1.56	0.12	<0.01
**receptive language**	**0.58**	**0.21**	**2.77**	**0.007**	**0.04**
**pre-test passive**	**−0.63**	**0.08**	**−7.74**	**<0.001**	**0.40**
short-term memory	24.46	17.46	1.40	0.16	0.02

model without receptive language multiple *R*^2^ = 0.38, adjusted *R*^2^ = 0.36. *F*(3,96) = 19.23, *p* < 0.001					
	estimate	SE	*t*	*P*	partial eta^2^
(intercept)	*−5.67*	*14.84*	*−0.38*	*0.70*	
**offline accuracy**	**33.56**	**10.94**	**3.07**	**0.003**	**0.008**
**pre-test passive**	**−0.59**	**0.08**	**−7.09**	**<0.001**	**0.35**
**short-term memory**	**38.97**	**17.22**	**2.26**	**0.03**	**0.05**

**Table 11.** Mediation analysis for the effect of offline accuracy on pre- to post-improvement.

	estimate	95% CI lower	95% CI upper	*p*
**average causal mediation effect**	**8.32**	**2.69**	**15.30**	**0.004**
average direct effect	5.66	−7.92	18.96	0.40
**total effect**	**13.98**	**1.20**	**27.03**	**0.04**
proportion mediated	0.60	0.13	2.88	0.04

## 4. Discussion

This study investigated whether children’s EF predicted their ability to revise interpretations during online processing and improve their comprehension of passives in the long term. Pre-registered analyses revealed no significant EF effects. The only significant effects were of receptive language and pre-test passive score on pre- to post-test passive comprehension improvement. Higher receptive language ability was associated with more improvement and higher pre-test scores were associated with less improvement (likely because higher pre-test scores meant less room for improvement). We conducted additional exploratory analyses based on observations about the reliability of the pre-registered eye movement measures and correlations between the predictors in the pre-registered models. These exploratory analyses revealed several effects that shed light on the potential relationships between EF and online processing and syntactic learning. We discuss each in turn below.

### 4.1. Relationship between executive function and online sentence processing

*A priori*, we had hypothesized that better EF could enable children to recover from initial misinterpretation more quickly and more often, resulting in quicker switches from the wrong to the right picture and higher revision accuracies during online processing. However, the pre-registered eye-tracking analysis procedures resulted in the elimination of a large number of trials and yielded poor to fair split-half reliability for the switch latency and revision accuracy measures. Widening the interval of interest resulted in significantly improved reliability. Exploratory analyses that included these more reliable online processing measures revealed additional effects compared to the pre-registered analyses.

First, we found that better EF was associated with higher revision accuracy after controlling for receptive language, pre-test scores and short-term memory (table 6: hypothesis 2 and figure 3). This is consistent with theoretical frameworks that have suggested that EF helps resolve competition between representations. On trials where children looked at the picture that matched the active sentence structure, better EF corresponded with a higher likelihood of switching to the correct picture that matched the passive sentence structure. Prior literature has provided considerable evidence for a link between EF and recovery from garden-pathing during the processing of syntactically ambiguous sentences in adults and some preliminary evidence for a similar link in children [15,16,19,20]. The present results corroborate and extend these findings by showing an association between EF and the processing of unambiguous sentences. For passive sentences that do not contain ambiguity *per se*, a probabilistic bias towards interpreting the first noun in the sentence as the agent could nevertheless result in initial misinterpretation. In these cases, better EF could assist in correcting the initial misinterpretation to arrive at the correct meaning.

In contrast to revision accuracy, the analyses of switch latency (i.e. how quickly children switched from the wrong to the right picture) did not reveal a relationship with EF after controlling for receptive language, pre-test scores and short-term memory. However, when receptive language, which showed a moderate correlation with EF, was excluded from the model, there was a significant EF effect after controlling for pre-test scores and short-term memory (table 8: hypothesis 1; figure 4). Mediation analysis found a significant total effect but not significant direct or indirect effects. Thus, additional studies are needed to determine whether EF influences the speed or efficiency of revision during online processing directly and/or via receptive language (see electronic supplementary material for relevant, additional results and discussion).

Together, the findings suggest that EF can influence the online processing of non-canonical structures like the passive. This effect cannot be explained by other confounds such as prior performance on passives and short-term memory. At least some of this influence also cannot be explained by receptive language skills, as we found with revision accuracy. It remains an open question whether other aspects of the relationship (e.g. with speed or latency) are mediated by receptive language. Thus, EF could influence online processing in one or more ways. Real-time recruitment of EF assists in revising misinterpretations, consistent with prior related evidence in adults and children. More indirectly, EF could promote language acquisition and receptive language broadly, which in turn could lead to some children being more efficient at language processing, but this possibility needs to be investigated further.

### 4.2. Relationship between executive function and syntactic learning

Going beyond in-the-moment effects, we considered the possibility that EF could impact longer-term syntactic learning because it enables revision during online processing, which can result in more accurate encoding and learning. The analyses that tested for an effect of online revision accuracy on improvement in passive comprehension from pre-test to post-test did not reveal any significant results when receptive language was either included or excluded from the model (table 7 and electronic supplementary material, Table 4). Thus, even though EF supports revision during passive processing, that in turn does not correspond to better longer-term learning of the passive structure.

We also tested whether EF *per se* predicted improvement in passive comprehension. These analyses showed that better EF predicted more improvement in passive comprehension (table 8: hypothesis 3a; figure 4) but that this effect was almost entirely mediated by receptive language (table 9: hypothesis 3a).

Finally, in post hoc analyses, we considered whether offline comprehension accuracy, unlike online revision accuracy, predicted pre- to post-improvement. There was a significant effect of this predictor when receptive language was not included in the model (table 10). Similar to the findings for EF as a predictor, this effect was almost entirely mediated by receptive language (table 11).

Overall, these findings suggest that EF’s effect on longer-term syntactic learning is distinct from its effect on online processing and is indirect, showing mediation by receptive language. We turn to discussion of the mediation findings next.

### 4.3. Mediation by receptive language

One clear finding from these results is that it is hard to disentangle effects of EF from effects of receptive language knowledge. A recurring theme in our analyses, especially for improvement from pre- to post-test, was that because of the high correlation between EF and receptive language, effects of EF were dependent on the presence/absence of receptive language in the model. We often found a significant effect of EF when the receptive language measure was excluded, but the effect became weaker and non-significant when this control variable was included. Mediation analyses did not reveal any significant direct effects of EF on the dependent variable and often revealed a significant indirect or mediated effect via receptive language. It is thus clear that the acquisition of receptive language knowledge and EF go hand in hand, such that it is not possible to separate their effects (though see [61]).

In these sorts of situations, there are (at least) three possible explanations [62]. The first possibility is that EF and language grow in parallel, but there is no causal relationship between the two. In the present case, this would imply that improvement in passive comprehension is related to a third variable that also independently causes changes in EF and receptive language. This third variable could be maturational (e.g. frontal lobe development) or environmental (language and other input) such that some children perform better than others across the board [63,64].

A second possibility is that EF causally influences children’s receptive language, which in turn leads to better learning of the passive structure. For example, better EF could correspond to more attentive encoding or more efficient processing of language input, or more sustained social interactions that result in higher quantity and/or quality of language input [65,66]. This could result in better receptive language and this higher language proficiency could scaffold further language learning, including better learning of the passive structure. Bootstrapping—the use of earlier language skills to acquire more complex language—is a known feature in children’s language acquisition, although the precise causal pathways are still disputed (see, e.g. [67,68]).

A third possibility is that better receptive language promotes EF and also promotes better learning of passives. For example, prior studies have suggested that gains in vocabulary may be related to growth in EF (e.g. [69]). Language can assist EF by enabling children to use inner speech to remember rules and stay on track. In support of this hypothesis, articulatory suppression can adversely affect EF performance in children under some conditions [70]. However, this is not true for all populations (e.g. autism spectrum disorder [71]). Further, double dissociations—good EF with poor inner speech and vice versa—attest to some degree of independence between language and EF [72].

Collectively, available evidence suggests a bidirectional and reciprocal relationship between EF and language in both children and adults [72,73]. Each could assist the other but may not be strictly necessary for the other. In the context of the present study, we found an EF effect on online revision accuracy after controlling for language and memory skills. This effect is consistent with multiple previous findings that suggest a process-specific role for EF in resolving competition between co-activated representations [9,21]. This role appears to be causal, as suggested by different training and adaptation paradigms [25–27]. Beyond that, our findings of linkages between EF, receptive language and syntactic learning are consistent with multiple possibilities, as discussed above. Although our study cannot conclusively distinguish between these possibilities, it is likely that all are at play. Language is not an isolated modular sub-system but is acquired in close interaction with developments in the broader social, cognitive and motor systems. Children’s improvements in one skill (e.g. EF) can lead to faster and more accurate language processing, which in turn can lead to better and more sophisticated language knowledge. This extra-linguistic knowledge can then enable faster and more accurate responses in EF tasks. Thus, a virtuous spiral of development may be present, with improvements in multiple systems mutually influencing one another.

### 4.4. Measurement challenges in individual differences studies

The success of individual differences studies like ours is dependent on reliable measurement, which is still somewhat of a challenge for cognitive variables like EF (for a general discussion see [74]. Notably, our pre-registered analyses showed little effect of EF on our outcome measures, but in many cases, the key variables (e.g. switch latency) did not meet the high levels of reliability required for individual differences studies. There are several possible reasons for this. One is the tension between experimental paradigms, which seek to reduce between-participant variance and consign it to measurement error, and the goal of individual differences research, which aims to maximize between-participant variability in order to rank individuals [48]. We note, however, that variables like switch latency measured via eye-tracking have yielded meaningful differences in language acquisition studies [52], although the psychometric nature of the task is rarely scrutinized (see [56]).

Another determinant of reliability is the amount of data over which reliabilities are computed. In our exploratory analyses, we computed new eye-tracking measures based on a wider window, which revealed more reliable measurements and suggested a role for EF in children’s language processing. The emergence of a positive relationship that is dependent on reliability is not unexpected; unreliable measures weaken any true relationship between the underlying constructs. The finding is similar to a recent longitudinal study investigating individual differences in language as predicted by measures of statistical learning (SL), reported in Kidd *et al*. [75]. They found that their SL measures increased in reliability as the children grew older, and only at later ages did the expected observed association between SL and language emerge. The finding of developmental differences in task reliability is an additional difficulty developmental studies must deal with, since the age of children influences their ability to participate in cognitive tasks, for instance, due to reduced attention span, which affects the amount and quality of data that can be collected. The creation of developmentally sensitive and psychometrically reliable tasks that measure skills such as EF, SL and language processing is important if we are to understand the functional importance of different skills and the relationships between them.

## 5. Conclusions and future directions

The present study adds to the emergent literature on how EF may impact language acquisition. We tested a substantially larger sample of children than previous studies, extended the enquiry to syntactically unambiguous sentences and investigated EF’s effects on processing as well as longer-term acquisition. For processing, the findings offer a relatively clear answer—EF can assist in recovery from the temporary misinterpretation of sentences during online processing. This effect is not restricted to special cases like garden-path sentences. It extends to unambiguous non-canonical sentence structures like the passive. For longer-term acquisition, the results are consistent with different causal pathways. Future studies can employ longitudinal designs that track children over several years—e.g. from ages two to five when both EF and language skills grow substantially—to determine which skills are primary and which arise as consequences. Our findings suggest that such designs might benefit from procedures for evaluating the reliability of different measures, employing repeated measurements of any given construct, aggregating across tasks where applicable and using multimodal techniques. More broadly, the results suggest that EF is a skill that is relevant for sentence processing in children, and the inclusion of this construct in theories and empirical studies of language acquisition can clarify both its utility and limits therein.

## Data Availability

All data and scripts are available on OSF [76]. Supplementary material is available online [77].

## Footnotes

^1^
In Dutch, the same verb forms can be used to indicate the simple present as well as the present progressive tense.

^2^
Example R code: model<-lm(latency ~ 1 + ExecutiveFunction + PreTest + LanguageProficiency + ShortTermMemory + ActiveSentenceLatency).

^3^
Because all IVs are between-participants individual difference characteristics, a mixed-effects model of trial-level data would be minimally different with only one additional term (by-item intercept). Therefore, we chose to go with multiple linear regression, the results of which can be interpreted more intuitively for the variance in the DV that can be explained by each IV.

^4^
This approach to measuring the speed of syntactic revision is analogous to the widely used method for measuring the speed of lexical processing (e.g. see [60] and references therein).

^5^
Model equations for mediation analysis for hypothesis 1: m1Mediator<-lm(ReceptiveLanguage ~ QuantileEFScore+ Pre.Passive + ShortTermMemory + ActiveTrialLatency, data=hyp1). m1Full<-lm(MeanLogLatency~QuantileEFScore+ Pre.Passive + ShortTermMemory + ActiveTrialLatency + ReceptiveLanguage, data=hyp1). results <- mediation::mediate(m1Mediator, m1Full, sims=2000, boot=TRUE, treat=‘QuantileEFScore’, mediator=‘ReceptiveLanguage’, control.value=1, treat.value=4).

^6^
Model equations for mediation analysis for hypothesis 3a: m3AMediator<-lm(ReceptiveLanguage ~ QuantileEFScore + Pre.Passive + ShortTermMemory, data=hyp3A). m3AFull<-lm(PostMinusPre ~ QuantileEFScore + Pre.Passive+ ShortTermMemory + ReceptiveLanguage, data=hyp3A). results <- mediation::mediate(m3AMediator, m3AFull, sims=2000, boot=TRUE, treat=‘BinaryEFScore’, mediator=‘ReceptiveLanguage’, control.value=1, treat.value=4).

^7^
Model equations for mediation analysis for the effect of offline accuracy: mBehMediator<-lm( ReceptiveLanguage ~ QuantileBehScore + Pre.Passive + ShortTermMemory, data=beh) mBehFull<-lm(PostMinusPre ~ QuantileBehScore + Pre.Passive + ShortTermMemory + ReceptiveLanguage, data=beh). results <- mediation::mediate(mBehMediator, mBehFull, sims=2000, boot=TRUE, treat=‘QuantileBehScore’, mediator=‘ReceptiveLanguage’, control.value = 1, treat.value=4).

## Appendix

Sentences used in pre- and post-test sessions.

**Table:**

trial #	set 1	set 1 English translation	set 2	set 2 English translation
1	het paard kietelt de aap	the horse is tickling the monkey	de leeuw wordt geverfd door de geit	the lion is being painted on by the goat
2	de beer huilt	the bear is crying	de aap zwemt	the monkey is swimming
3	de koe wordt gekieteld door de aap	the cow is being tickled by the monkey	de muis wordt gekieteld door het schaap	the mouse is being tickled by the sheep
4	het paard wordt geverfd door de beer	the horse is being painted on by the bear	de leeuw huilt	the lion is crying
5	de aap huilt	the monkey is crying	het schaap wordt geschopt door de aap	the sheep is being kicked by the monkey
6	het schaap trekt de geit	the sheep is pulling the goat	de beer zwemt	the bear is swimming
7	de koe zwaait	the cow is waving	het schaap slaapt	the sheep is sleeping
8	de beer wordt getrokken door de geit	the bear is being pulled by the goat	de muis kietelt de geit	the mouse is tickling the goat
9	de leeuw schopt het paard	the lion is kicking the horse	het paard schopt de leeuw	the horse is kicking the lion
10	de muis trekt de koe	the mouse is pulling the cow	de muis zwaait	the mouse is waving
11	de geit slaapt	the goat is sleeping	de geit wordt getrokken door de beer	the goat is being pulled by the bear
12	de muis wordt geschopt door het paard	the mouse is being kicked by the horse	de leeuw verft de aap	the lion is painting on the monkey
13	de leeuw zwemt	the lion is swimming	de geit zwaait	the goat is waving
14	de beer schopt het schaap	the bear is kicking the sheep	de beer wordt geverfd door het paard	the bear is being painted on by the horse
15	de leeuw wordt getrokken door de koe	the lion is being pulled by the cow	de koe trekt de muis	the cow is pulling the mouse
16	de geit kietelt de muis	the goat is tickling the mouse	de aap kietelt het paard	the monkey is tickling the horse
17	de aap wordt geschopt door het schaap	the monkey is being kicked by the sheep	de koe wordt getrokken door de leeuw	the cow is being pulled by the lion
18	de muis slaapt	the mouse is sleeping	het schaap schopt de beer	the sheep is kicking the bear
19	de koe verft de beer	the cow is painting on the bear	de koe slaapt	the cow is sleeping
20	het schaap wordt gekieteld door de muis	the sheep is being tickled by the mouse	de geit trekt het schaap	the goat is pulling the sheep
21	de geit wordt geverfd door de leeuw	the goat is being painted on by the lion	de beer verft de koe	the bear is painting on the cow
22	het paard zwemt	the horse is swimming	het paard wordt geschopt door de muis	the horse is being kicked by the mouse
23	de aap verft de leeuw	the monkey is painting on the lion	de aap wordt gekieteld door de koe	the monkey is being tickled by the cow
24	het schaap zwaait	the sheep is waving	het paard huilt	the horse is crying

Sentences used in the exposure session. P = practice. For list 2, the critical sentences changed from actives to passives and vice versa.

**Table:**

trial #	list 1	list 1 English translation
P	de poes met de wanten kust de vos	the cat with the mittens kisses the fox
P	de hond met de sokken slaat de kip	the dog with the socks hits the chicken
P	de eend met de schoenen knuffelt de uil	the duck with the shoes hugs the owl
1	de leeuw met de trui wordt gewassen door het schaap	the lion with the sweater is washed by the sheep
2	het paard met de hoed draagt de aap	the horse with the hat carries the monkey
3	de beer met de trui wordt geduwd door het schaap	the bear with the sweater is pushed by the sheep
4	de aap met de broek wordt gewassen door de geit	the monkey with the pants is washed by the goat
5	de muis met de jurk draagt het schaap	the mouse with the dress carries the sheep
6	de geit met de trui vangt de beer	the goat with the sweater catches the bear
7	de muis met de sjaal wordt geduwd door de leeuw	the mouse with the scarf is pushed by the lion
8	het schaap met de strik aait het paard	the sheep with the bow pets the horse
9	de beer met de jurk wast de muis	the bear with the dress washes the mouse
10	het paard met de broek wordt geduwd door de geit	the horse with the pants is pushed by the goat
11	de leeuw met de hoed aait de beer	the lion with the hat pets the bear
12	de muis met de strik wordt gedragen door de geit	the mouse with the bow is carried by the goat
13	de leeuw met de jurk vangt de koe	the lion with the dress catches the cow
14	de muis met de trui wordt gekamd door het paard	the mouse with the sweater is combed by the horse
15	de aap met de hoed wordt geaaid door de beer	the monkey with the hat is petted by the bear
16	het schaap met de sjaal vangt de muis	the sheep with the scarf catches the mouse
17	de geit met de jurk aait de koe	the goat with the dress pets the cow
18	het paard met de sjaal wordt gekamd door de beer	the horse with the scarf is combed by the bear
19	de muis met de hoed duwt de aap	the mouse with the hat pushes the monkey
20	het paard met de strik wast de beer	the horse with the bow washes the bear
21	de geit met de sjaal wordt geaaid door de muis	the goat with the scarf is petted by the mouse
22	de leeuw met de broek kamt het schaap	the lion with the pants combs the sheep
23	de beer met de hoed wordt gedragen door de aap	the bear with the hat is carried by the monkey
24	de muis met de broek wast het paard	the mouse with the pants washes the horse
25	de koe met de trui wordt gedragen door het schaap	the cow with the sweater is carried by the sheep
26	de beer met de broek duwt de geit	the bear with the pants pushes the goat
27	de aap met de sjaal aait de leeuw	the monkey with the scarf pets the lion
28	de koe met de hoed wordt gekamd door de muis	the cow with the hat is combed by the mouse
29	de geit met de broek wordt gevangen door het paard	the goat with the pants is caught by the horse
30	de koe met de strik duwt de muis	the cow with the bow pushes the mouse
31	het schaap met de broek wordt gewassen door de aap	the sheep with the pants is washed by the monkey
32	de leeuw met de strik wordt gevangen door de muis	the lion with the bow is caught by the mouse
33	de koe met de broek draagt de geit	the cow with the pants carries the goat
34	de leeuw met de sjaal wordt geaaid door het paard	the lion with the scarf is petted by the horse
35	het schaap met de trui kamt de aap	the sheep with the sweater combs the monkey
36	het paard met de jurk wordt gedragen door de leeuw	the horse with the dress is carried by the lion
37	de beer met de strik wordt gekamd door de koe	the bear with the bow is combed by the cow
38	het paard met de trui duwt het schaap	the horse with the sweater pushes the sheep
39	de aap met de jurk wordt gevangen door de koe	the monkey with the dress is caught by the cow
40	de geit met de strik wordt gewassen door de leeuw	the goat with the bow is washed by the lion
41	de beer met de sjaal draagt de koe	the bear with the scarf carries the cow
42	de aap met de strik vangt het paard	the monkey with the bow catches the horse
43	het schaap met de jurk wordt geaaid door de koe	the sheep with the dress is petted by the cow
44	de geit met de hoed kamt de leeuw	the goat with the hat combs the lion
45	de koe met de jurk wordt geduwd door de aap	the cow with the dress is pushed by the monkey
46	het schaap met de hoed wordt gevangen door de beer	the sheep with the hat is caught by the bear
47	de koe met de sjaal wast de leeuw	the cow with the scarf washes the lion
48	de aap met de trui kamt de geit	the monkey with the sweater combs the goat
